# Suppressive effect of glycyrrhizic acid against lipopolysaccharide-induced neuroinflammation and cognitive impairment in C57 mice via toll-like receptor 4 signaling pathway

**DOI:** 10.29219/fnr.v63.1516

**Published:** 2019-04-29

**Authors:** Wenfeng Liu, Shun Huang, Yonglian Li, Kun Zhang, Xi Zheng

**Affiliations:** 1 School of Biotechnology and Health Sciences, Wuyi University, Jiangmen, China; 2 International Healthycare Innovation Institute, Jiangmen, China; 3 Nanfang PET Center, Nanfang Hospital, Southern Medical University, Guangzhou, China; 4 School of Eco-environment Technology, Guangdong Industry Polytechnic, Guangzhou, China; 5 Susan Lehman Cullman Laboratory for Cancer Research, Department of Chemical Biology, Ernest Mario School of Pharmacy, Rutgers, The State University of New Jersey, Piscataway, NJ, USA

**Keywords:** glycyrrhizinic acid, LPS, neuroinflammation, toll-like receptor (TLR4), Alzheimer’s disease

## Abstract

**Background:**

Glycyrrhizinic acid (GA), a major active ingredient enriched in the roots of licorice, possesses well-confirmed anti-inflammatory effects.

**Objective:**

To evaluate the underlying mechanisms of GA against lipopolysaccharide (LPS)-induced chronic neuroinflammation and memory impairment.

**Design:**

We explored to investigate the effects of GA on neuroinflammation and memory impairment in an LPS-induced Alzheimer’s mouse model.

**Results:**

Data of micro-PET/CT imaging and morris water maze test suggested that GA, when administrated orally, could reverse LPS-induced abnormalized glucose intake and metabolism in the brain and alleviate LPS-induced memory loss and cognitive defects in mice. Histological and immunohistochemical staining results revealed that GA treatment suppressed overexpressions of pro-inflammatory cytokines of IL-1 β and TNF-α in the brain of C57 mice by inhibiting toll-like receptor 4 (TLR4) signaling pathway activation.

**Conclusion:**

Our findings suggest that GA may be a therapeutic agent for the treatment of neuroinflammation and cognitive impairment.

## Popular scientific summary

- Oral administration of GA could dose-dependently reverse LPS-induced normalized glucose intake and metabolism in the brain and alleviate LPS-induced memory loss and cognitive defects in mice. This may be mainly due to the decrease of pro-inflammatory cytokines.
- GA at two different doses further regulated the over-expression of pro-inflammatory cytokines via toll-like receptor 4 signaling pathway related to neuro-inflammation, resulting in the inflammatory response alleviation and memory loss and cognitive defects risk reduction. Hence, GA could alleviate LPS-induced normalizedglucose intake and pro-inflammatory cytokines, which most closely connected with Alzheimer’s disease.

## 

Alzheimer’s disease (AD) is a multi-factorial neurodegenerative disorder, which has been regarded as one of the most life-threatening diseases among the aged (1). Although the complicated mechanism of AD is not yet clearly understood, the significant role of neuroinflammation, which refers to inflammation in the brain, has drawn much attention in accumulating studies (2, 3). Evidence is accumulating to suggest that under neuroinflammatory conditions, microglia activated extensively, and consequently upregulating expressions of pro-inflammatory mediators, that further result in astrocyte activation and neuronal damage in the pathophysiology of AD (4, 5). In addition, neuroinflammation stimulated by activated stimulus, such as β-amyloid (Aβ) aggregation and α-synuclein proteins, leads to the overexpression of pro-inflammatory cytokines, including interleukin 1 β (IL-1β) and tumor necrosis factor α (TNF-α), which can in turn induce microglial activation, producing a cycle of neuroinflammation (6, 7). Furthermore, sustained neuroinflammatory processes expedite an injury of brain tissue, which is ultimately linked with the development of behavioral pathology (8). Therefore, the inhibition of excessive pro-inflammatory cytokines production may be proposed as a therapeutic strategy for neuroinflammation and cognitive impairment, which contribute to AD progression.

**Fig. 1:**
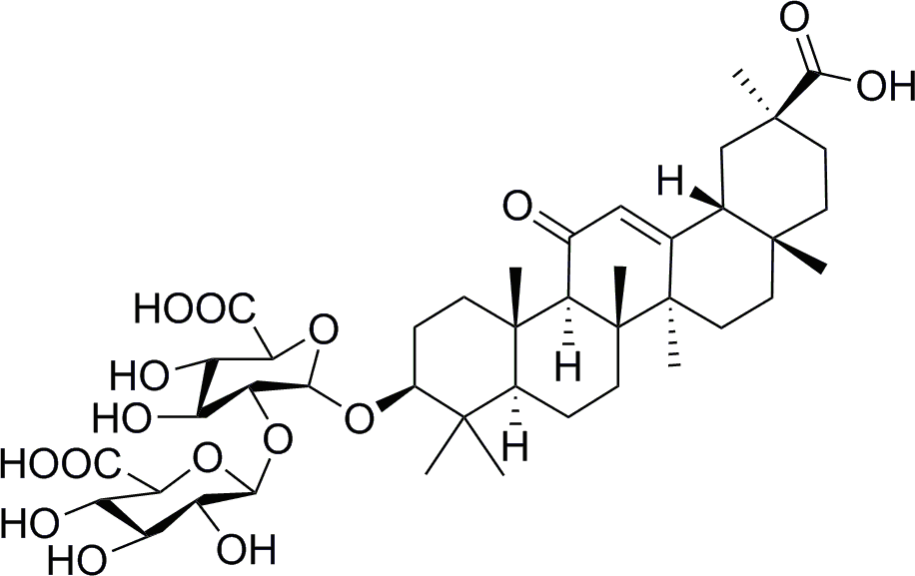
Chemical structures of GA.

Licorice, a flowering plant, has been widely used as a natural sweetener and traditional folk herbal medicine (9, 10). It was indicated that the root of licorice, which confirms to have beneficial effects on the brain, currently uses as an antidepressant (11) and memory enhancing agent (12). Furthermore, licorice extract has been confirmed to inhibit Aβ secretion and improve cognitive deficits in AD mode (12). Accumulating evidence suggests that glycyrrhizic acid (GA, Fig. 1), derived from the root of licorice, shows a low risk of side effects and possesses varieties of bioactivities, such as antioxidant (13), antitumor (14), and anti-inflammatory activities (15). Extensive evidence indicates that GA affords robust neuroprotection in the postischemic brain *via* its anti-inflammatory properties against high-mobility group box 1 (HMGB1) phosphorylation and suppresses the inductions of inflammatory cytokines (16). Similarly, it is demonstrated that the GA-mediated neuroprotection was accompanied with the suppression of the glutamate-induced neuronal death (17). Moreover, recent studies have indicated that GA could be served as an anti-inflammatory agent in kainic acid-induced microglia (18). Importantly, there are several reports that suggest that GA inhibits the overexpressions of pro-inflammatory cytokines and the activations of various transcription factors in LPS-treated cells (19, 20). However, studies addressing the effects of GA on LPS-induced neuroinflammation and cognitive impairments and the anti-neuroinflammatory mechanisms of action of GA *in vivo* are still lacking.

In this study, a lipopolysaccharide (LPS)-induced Alzheimer’s mouse model was employed to evaluate the potential effects of GA on chronic neuroinflammation and memory impairment. Furthermore, we investigated the neuroprotective mechanisms of GA to gain some new insights into the therapeutic strategies for neuroinflammation and Neurodegenerative disorder.

## Materials and Methods

### Materials

Glycyrrhizinic acid was purchased from J&K Scientific Co. (Shanghai, China). LPS (*Escherichia coli* O55:B5) was obtained from Sigma-Aldrich (St. Louis, MO, USA). TTP488, synthesized using published procedures that were described in patent WO 2011/041198 A1, was used as a standard compound. Antibodies against TLR4, Myd88, MAPK p38, NF-κB p65, TNF-α, and IL-1β were purchased from Bioss Biotechnology Co. (Beijing, China).

### Animals and drug treatment

Female C57 mice (4–5 weeks old) were purchased from the Center of Animal Test of Southern Medical University and Nanfang Hospital (SCXK/20160041, Guangzhou, China). All animals were divided into groups and housed (25±1°C at 50% relative humidity) under a 12 h/12 h light–dark cycle conditions. Food and water could be accessed *ad libitum*. All animal care and experimental procedures were carried out in strict accordance with the National Institute of Health’s Guide for the Care and Use of Laboratory Animals. At the start of the experiment, the animals weighed 22–25 g and were 8–9 weeks old.

As shown in previous publication (21), we established the LPS-induced Alzheimer’s disease model. Furthermore, the animals were randomly divided into five groups, with eight mice in each group:

Control group: Mice of this group were injected with phosphate-buffered saline (PBS) once daily for 14 days.

LPS group: Mice of this group were injected with PBS once daily for 7 days. After 7 days, the mice were administered intraperitoneally (i.p.) with LPS (250 μg/kg) once daily for 7 days.

TTP488 group: Mice of this group were injected with TTP488 (5 mg/kg) once daily for 7 days. After 7 days, the mice were administered i.p. with LPS (250 μg/kg) once daily for 7 days.

GA 50 group: Mice of this group were injected with GA (50 mg/kg) once daily for 7 days. After 7 days, the mice were administered i.p. with LPS (250 μg/kg) once daily for 7 days.

GA 100 group: Mice of this group were injected with GA (100 mg/kg) once daily for 7 days. After 7 days, the mice were administered i.p. with LPS (250 μg/kg) once daily for 7 days.

### Micro-PET imaging

The method, used for micro-positron emission tomography (micro-PET) imaging of mice, was established as described in previous publication (21). After 2 weeks of drugs administration, the micro-PET was conducted to investigate inhibitory effects of GA on brain glucose uptake in mice from all groups. Food was removed from the mouse cage 12 h before this experiment. The animals were anesthetized with 7% chloral hydrate and then injected i.p. with ^18^F-fluordeoxyglucose (^18^F-FDG). Sixty minutes after ^18^F-FDG injections, the mice were scanned by using an Inveon Micro-PET/CT system (Siemens Healthiness, Munich, Germany). After PET acquisition, the micro-PET images were reconstructed using the micro-PET/CT manager (Siemens Medical Solutions Inc., Pittsfield, USA). These whole-body images of the mice from all groups, reconstructed by using the micro-PET/CT manager, were used for analysis. The region-of-interest was drawn guided by PET images, and tracer uptake was measured using the software of Inveon Research Workplace. Quantification of the ^18^F-FDG uptake in mouse brains was performed to obtain the maximum standardized uptake value (SUVmax) and mean standardized uptake value (SUVmean).

### Morris water maze (MWM) test

After 2 weeks of drug administration, the MWM test was conducted to evaluate the learning and memory abilities in mice from all groups (22). The water maze consisted of a plastic pool (120 cm in diameter) filled with opaque water at 24 ± 1°C and a hidden platform (10 cm in diameter). An overhead video camera connected to the SMART video-tracking and analysis system (TSE, Bad Homburg, Germany) was used to track the movements of mice and record all trials. In the navigation test, the hidden platform (1 cm under the water) was kept constant in the middle of one certain quadrant (Target quadrant) throughout training. The training consisted of four sessions of trials (one session/day, one trial/session). Each trial was performed for 60 sec until the mouse climbed onto the hidden platform target, and the escape latency of the mouse was recorded. After the navigation test, the spatial probe test was carried out without the platform in the quadrant. Each mouse was put into the pool and swam for 120 sec. The percentage (%) time spent in each quadrant and the number of times the mice crossed the platform area were measured using the SMART video-tracking and analysis system

### Nissl staining

Female C57 mice were all sacrificed after the MWM test. Brains of all groups were removed *in toto*, washed twice with 0.01 M PBS, and then fixed in 4% paraformaldehyde (PFA). Paraffin-embedded brain specimens were placed in a rotary microtome, and 4-μm-thick consecutive coronal slices were prepared until the hippocampal surface was reached. Tissue slices were cleared in xylene for 5 min, dehydrated in a series of graded ethanol (100 and 75%), and consequent slices were stained with a warmed 0.5% cresyl violet solution for 5 min, rinsed with distilled water. Images were captured using Olympus confocal microscope (IX-73, Tokyo, Japan). Normal neurons were quantified at 400× magnification. The normal neurons had prominent Nissl granules and a light, distinct nucleus. The analysis of normal neuronal cells was performed on three nonoverlapping fields per slice.

### Immunohistochemistry staining

*In situ* expression of pro-inflammatory cytokines (IL-1β and TNF-α) and transcription factors (NF-κB p65, MAPK p38, myd88, and TLR4) in brain were performed as follows. Serial 4-μm tissue sections were immersed in xylene (thrice for 15 min at room temperature), rehydrated in 100% ethanol (twice for 5 min at room temperature), 85% ethanol (for 5 min), and 75% ethanol (for 5 min), and then rinsed with distilled water. Antigen retrieval was performed by boiling slices for 30 min in citrate antigen retrieval solution, pH = 6.0. The slides were blocked in 3% hydrogen peroxide, 3% bovine serum albumin (BSA), and 0.25% TX-100 in PBS for 1 h at room temperature, and subsequently incubated overnight at 4°C with the primary antibodies (anti-IL-1β, anti-TNF-α, anti-NF-κB p65, anti-MAPK p38, and anti-myd88: 1:600; and anti-TLR4: 1:700). Sections were washed thrice with PBS (pH = 7.4) and were subsequently incubated with goat anti-rabbit antibody. The sections were then counterstained with hematoxylin for 3 min to stain cell nuclei. Moreover, the sections, labeled to detect pro-inflammatory cytokines (IL-1β and TNF-α) and transcription factors (NF-κB p65, MAPK p38, myd88, and TLR4) positive cells, were visualized using a microscope.

### Statistical analysis

Quantitative data are expressed as mean ± standard deviation (SD). Statistical comparisons of the data and the statistical signifcance were determined by using the SPSS 19.0 analysis of variance (ANOVA) test and Tukey’s post hoc test. *P* values of < 0.05 were considered statistically significant.

## Results

### Effects of GA on LPS-induced cognitive impairment and related cerebral metabolism in C57 mice

**Fig. 2:**
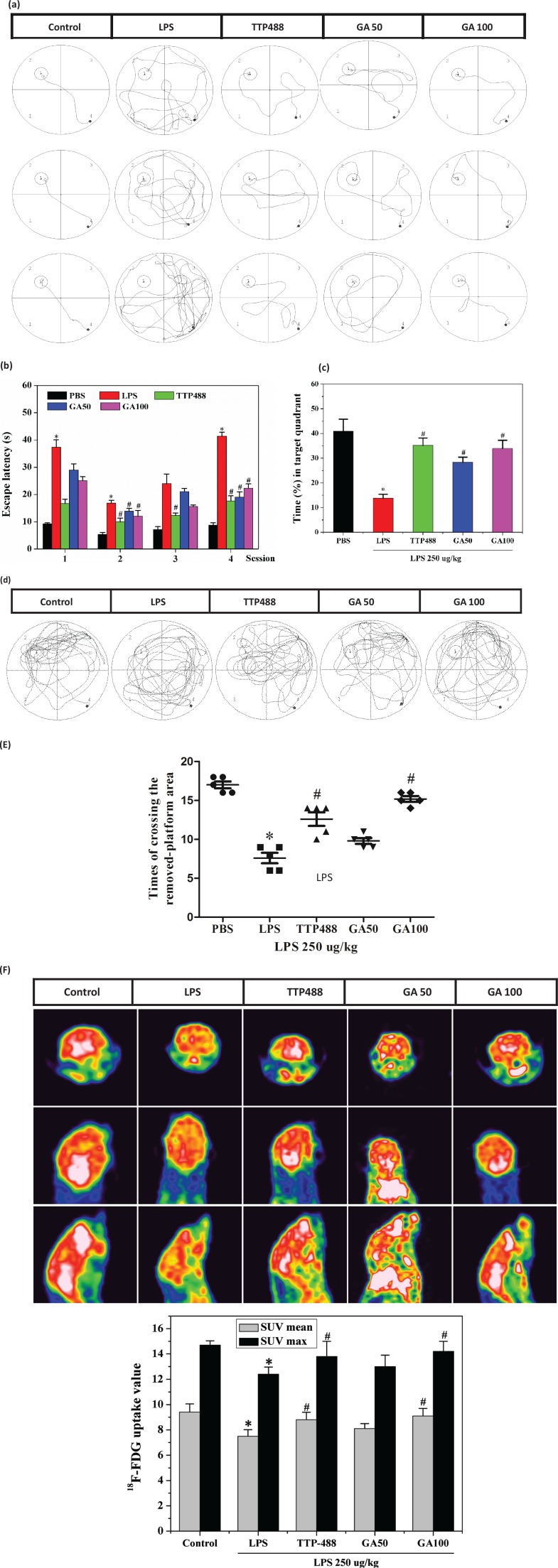
Effects of GA on LPS-induced memory impairment and related cerebral metabolism. (a) Movement tracks of mice in the place navigation (hidden platform) test. (b) The escape latencies of mice in the place navigation (hidden platform) test. (c) The percentage of time the mice spent in searching for the target quadrant in the spatial probe test. (d) Movement tracks of mice in the spatial probe test. (e) The number of times the mice crossed the removed-platform area in the spatial probe test. (F) PET-CT images and ^18^F-FDG uptake of mouse brains. Data represent the mean ± SD (*n* = 8 mice per group). The significance of differences from control group is at *P < 0.05, from LPS group at #*P* < 0.05. GA: glycyrrhizinic acid.

LPS, when administrated systemically, has been reported to cause memory impairment and learning disability (23). Consequently, we investigated whether GA has any influence on LPS-induced memory impairment and learning disability using MWM. As shown in the place navigation (hidden platform) test (Fig. 2b), LPS infusion caused a significant disruption of learning and memory, indicated by an increase in the escape latency compared to the control group (*P* < 0.05). A post hoc analysis demonstrated that GA remarkably decreased the escape latency when administered i.p. with 50 mg/kg and 100 mg/kg in the second session (*P* < 0.05) and fourth session (*P* < 0.05) in LPS-treated mice. In addition, TTP488 significantly decreased the escape latency of the LPS-treated mice when given at 5 mg/kg during the second session (*P* < 0.05), third session (*P* < 0.05), and fourth session (*P* < 0.05) compared to control group. Movement tracks of the mice were in accordance with the escape latencies, GA50 (50 mg/kg) and GA100 (100 mg/kg) groups traveled less distance to arrive the platform (Fig. 2a).

In the spatial probe test, the hidden platform was removed, and the time spent in the target quadrant and the number of times mice from all groups crossed the target platform were assessed. As shown in Fig. 2c and 2d, compared with the control group, LPS group indicated a significant deficit in cognition, as shown by a longer latency time to the platform, whereas this result was reversed under TTP488 and GA treatment. These results indicated that TTP488 group and GA group mice spent more time in the target quadrant than the LPS group and had greater number of platform crossings. Furthermore, GA exhibited significant increases in time spent in the target quadrant and crossing times in a dose-dependent manner. In addition, the recorded tracks of mice confirmed these results (Fig. 2e).

LPS is a well-studied immunostimulator that induced immune neuroinflammatory responses that lead to cognitive impairment (23). Furthermore, LPS is reported to abnormalize glucose metabolism in the brain (24). Hence, to investigate the relevance of cerebral metabolism to cognitive impairment in mice, we determined the effects of GA at different doses on LPS-induced brain glucose uptake by using an Inveon small animal PET scanner. As described in Fig. 2f, after LPS injection, ^18^F-FDG uptake intensity in LPS group showed significant decrease in the brain, while the control group stayed at the high uptake level. It was confirmed that LPS abnormalized glucose metabolism in the brain. Moreover, ^18^FDG-PET imaging revealed that GA, when administrated orally, dose-dependently increased ^18^F-FDG uptake in the brain. In addition, GA100 (LPS+100 mg/kg/d) showed the same high uptake level in accordance with TTP488 (LPS+5 mg/kg/d), which was an antagonist of receptor for advanced glycation end products. These results indicated that GA can increase neuronal glucose utilization, keep normalized cerebral metabolism, and improve brain functions.

### Preventive effects of GA on LPS-induced neuron damage

**Fig. 3:**
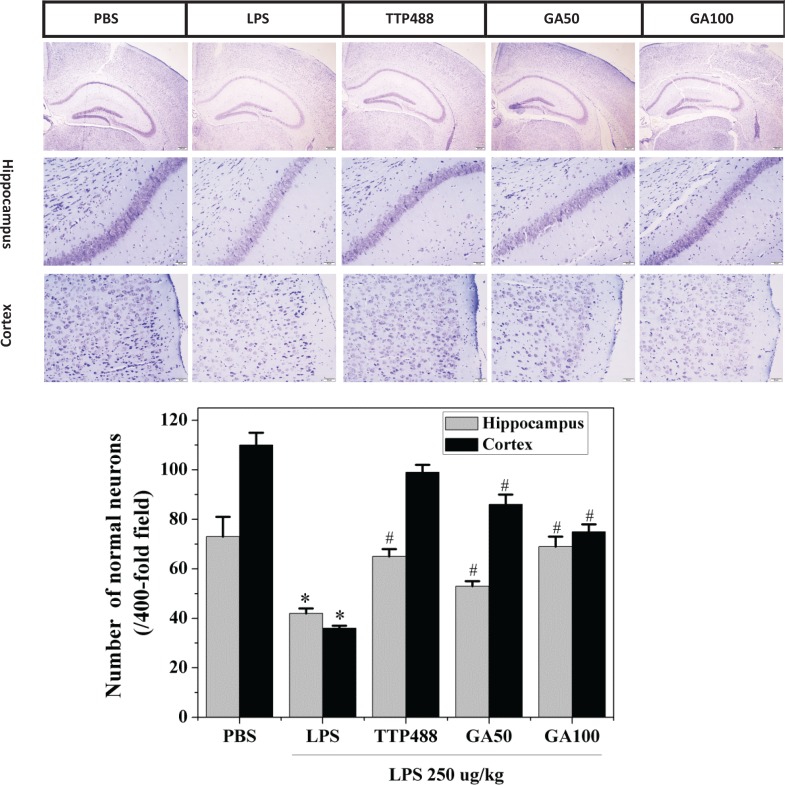
Effects of GA on LPS-induced neuron damage. Nissl staining and normal neuronal counts in the hippocampus and cortex of each group, scale bar = 50 μm. Data represent the mean ± SD (*n* = 8 mice per group). The significance of differences from control group is at *P < 0.05, from LPS group at #*P* < 0.05. GA: glycyrrhizinic acid.

The neuroprotective effect of GA on histopathologic changes was investigated by using Nissl staining. Nissl staining showed that LPS decreased neuronal viability compared to the control group. Histology of the cortex and hippocampus indicated that LPS treatment induced a remarkable loss of Nissl substances in cells and increased the level of damaged, fragmented, or dead neurons, whereas GA plus LPS administration exerted a dose-dependent neuroprotective effect, increasing the number of Nissl-stained cells and normal neurons in the hippocampus, compared with the LPS group mice (Fig. 3).

### Effects of GA on LPS-induced neuroinflammatory response in cortex and hippocampus

**Fig. 4:**
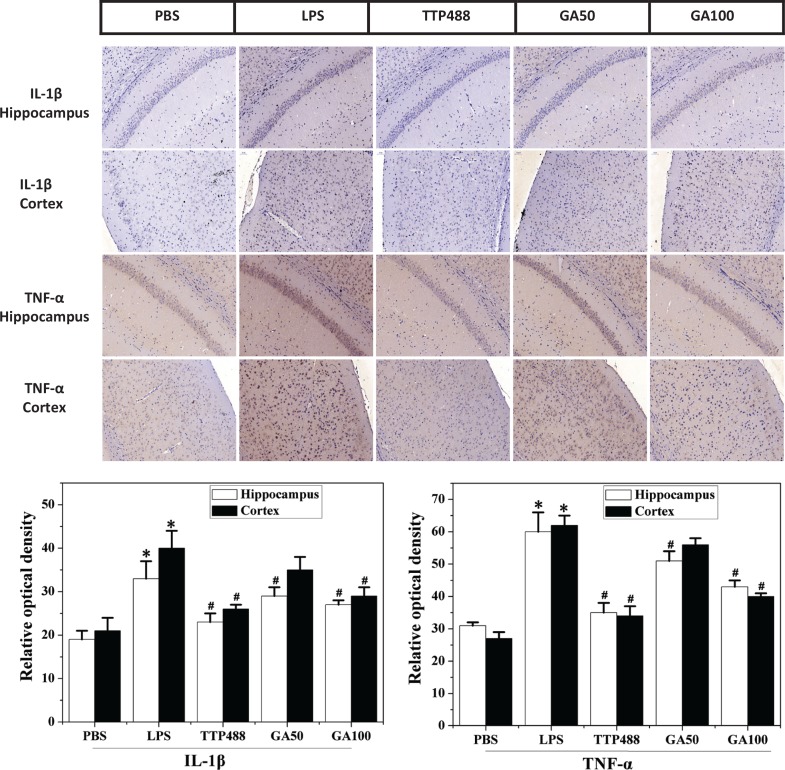
Immunostaining of GA against LPS-induced IL-1β and TNF-α expressions in the cortex and hippocampus of C57 mice, scale bar = 50 μm. The images (four sections per animal) were captured and quantified using the Image-Pro Plus software package. The significance of differences from control group is at *P < 0.05, from LPS group at #*P* < 0.05. GA: glycyrrhizinic acid. Data represent the mean ± SD (*n* = 8 mice per group).

The increased neuroinflammatory cytokine expressions are indices of microglial activation and lead to neuroinflammation and cognitive disorder in the brain (25). Thus, we used immunohistochemical techniques to examine whether GA affected LPS-induced expression of IL-1β and TNF-α in the brain. As described in Fig. 4, in comparison with the control group, a marked elevation in LPS-induced TNF-α and IL-1β expression was noted both in the hippocampus and cortex, respectively. compared with the control group. Pretreatment with 50 mg/kg of GA markedly suppressed IL-1β and TNF-α expressions , and it was further suppressed by 100 mg/kg GA (Fig. 4b). These results indicated that LPS-induced pro-inflammatory cytokine productions in the hippocampus of brains were dose-dependently suppressed by GA.

### Suppressive effects of GA on the TLR4 signaling pathway in LPS-induced neuroinflammation

**Fig. 5:**
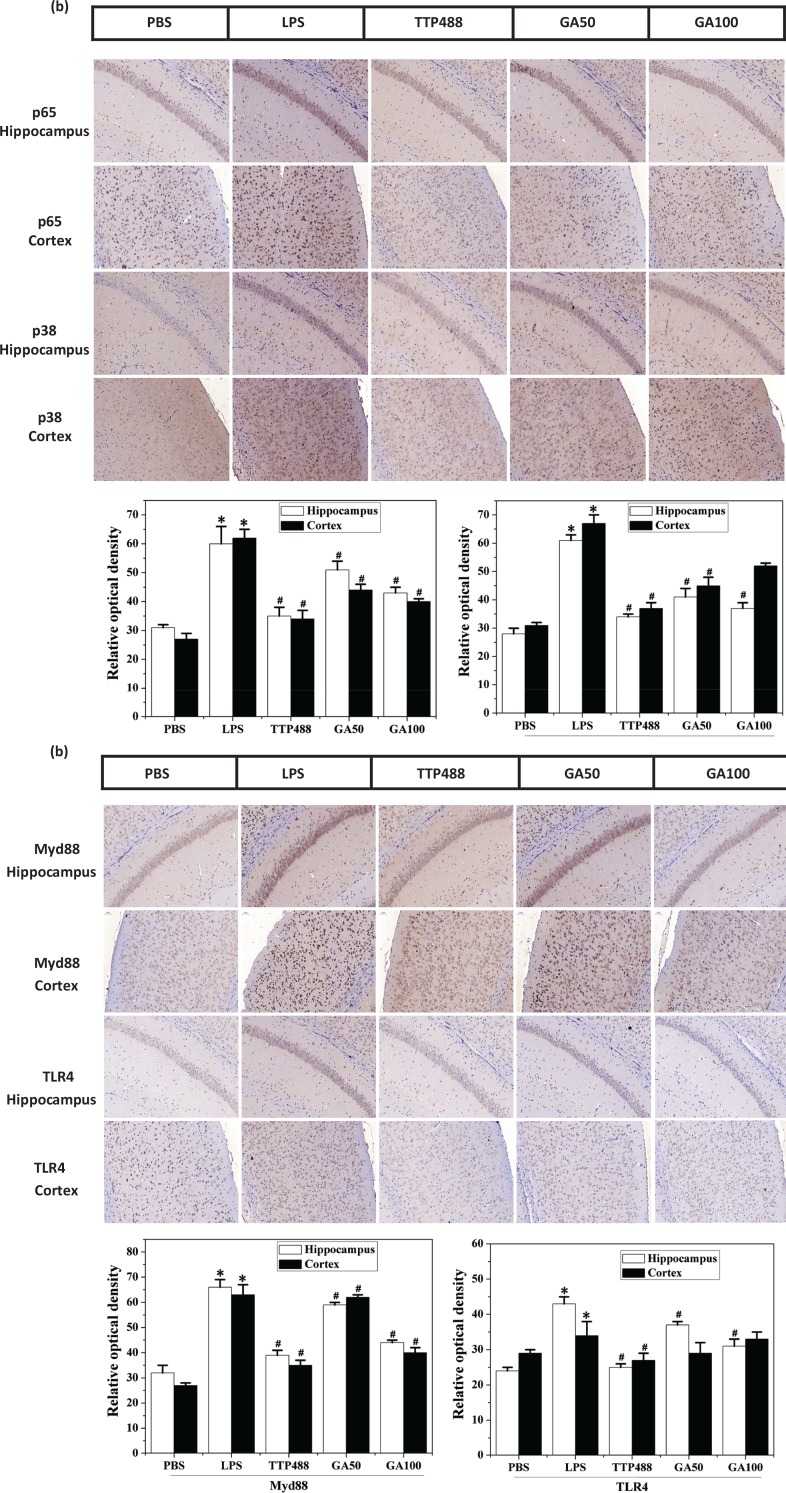
Anti-inflammatory mechanism of GA against LPS-induced neuroinflammation in mice brains. (a) Immunostaining of GA against LPS-induced NF-κB p65 and MAPK p38. (b) Immunostaining of GA against LPS-induced Myd88 and TLR4 expressions in the cortex and hippocampus of C57 mice, scale bar = 50 μm. The images (five sections per animal) were captured and quantified using the Image-Pro Plus software package. The significance of differences from control group is at *P < 0.05. GA: glycyrrhizinic acid.

TLR4 signaling cascade, recruited and interacted with Myd88 that resulted in the MAPK and NF-κB activation upon LPS administration, has been reported to be highly associated with the response of neuroinflammation and involved in the development of AD and other Neurodegenerative disorder (26). Hence, to further investigate the neuroprotective mechanisms of action of GA, TLR4, a key signaling transduction factor modulating a wide range of neuroinflammatory cytokines and various transcription factors expression, was examined by immunohistochemical techniques. As illustrated in Fig. 5a, treatment with GA dose-dependently improved the overexpressions of p38, p65, myd88, and TLR4 as compared to LPS group, suggesting that GA suppressed LPS-induced IL-1β and TNF-α activation by inhibiting TLR4 signaling pathway.

## Discussion

AD is one of several Neurodegenerative disorder that is closely related to aging, which is a major cause of gradual declines in brain function and is implicated in cognitive disorders, memory loss, and dementia (27). Accumulating evidence revealed that systemic injection of low-dosage LPS in mice induces a spectrum of behavioral changes, including impaired spatial learning, which well mimic the symptoms of AD patients (23). Activation of α-, β-, and γ-secretases by systemic LPS elevates Aβ42 expression and amyloidogenesis, respectively, and thus triggers the secretion of various pro-inflammatory cytokines in the brain, which ultimately lead to physiological and behavioral symptoms via neuroimmune interactions (28, 29).

Interestingly, a number of studies have revealed that changes in global and regional brain metabolism and energetics play an important role in the pathogenesis of cognitive impairment, making it a promising target for therapeutic intervention to restore cognitive behaviors. Furthermore, it was suggested that late-onset AD showed decreased bilateral glucose metabolism, particularly in the temporal and parietal regions (30). In this article, we determined the effects of GA on LPS-induced neuroinflammation and memory impairment in LPS-induced Alzheimer’s mouse model. Moreover, the effects of GA on cerebral glucose metabolism were also tested by micro-PET imaging. The behavioral experiments showed that GA could attenuate LPS-induced cognitive impairment, including working memory and spatial cognitive memory. These results also showed that the administration of GA i.p. considerably restored the brain glucose metabolism impaired by repeat LPS injections, indicating the improving effects of GA on brain functions in C57 mice.

Numerous studies have revealed that the hippocampus and cortex are involved in cognitive and behavioral functions, which are widely disturbed in AD (31). Furthermore, it is indicated that alteration of the structure and function of the pyramidal cells in the hippocampus and cortex is associated with learning and memory abilities of the mice (32). Hence, we investigated whether the normally pyramidal cells were damaged and decreased in the LPS-induced mice brains by using Nissl staining. The histological results indicated that LPS injection resulted in the damage of pyramidal cells in the hippocampus and cortex, which was characterized by a considerable loss of Nissl-stained cells compared with control group mice. However, GA treatment reversed this pathological change in the hippocampus. We found that GA exerted a dose-dependent preventive effect against the damage of pyramidal cells, which, in turn, improved the cognitive and behavioral functions of mice brains.

It is noteworthy that neuroinflammation, induced by LPS administration, is one of the causes of the neuropathogenesis and cognitive impairment (33). Previous studies have indicated that the release of pro-inflammatory cytokines and neuronal death are strongly correlated with increases in the severity of the afflicted dementia (34). Moreover, it was reported that injection of LPS activated glial cells to synthesize and secrete IL-1β and TNF-α (35), which transferred into the brain to induce the synthesis of pro-inflammatory transcription factors, producing a persistent neuroinflammation (36). In addition, systematic administration of LPS had been usually used in experimental models of neuroinflammation (37). In this study, we evaluated the effects of GA on the LPS-induced inflammatory processes. Our observations demonstrated that administration of LPS i.p. significantly exacerbated inflammatory responses in the brain through the release of IL-1β and TNF-α. Moreover, GA dose-dependently decreased neuron damage and significantly attenuated the overexpressions of inflammatory cytokines in the hippocampus of the brain.

Recent studies have demonstrated that microglial cells are the resident macrophage-like population within the central nervous system (CNS), and they are recognized as the prime component of the brain immune system (38). Microglia detect invading pathogens via various receptors, including TLR4 (together with CD14 and MD2 in microglia) (25, 39), which are expressed on glial cell surface. In particular, LPS is well known as a TLR4 ligand (36). Once microglia cells are activated by LPS, the interaction of LPS with TLR4 would trigger a second MyD88-dependent pathway, and thus would result in the initiation of a series of inflammatory cascade, including MAPK, as well as NF-κB signaling pathway. The activation of these signal transduction pathways promoted the overexpression of pro-inflammatory cytokines, contributing to the neuronal damage in various Neurodegenerative disorder (40, 41). This suggests that TLR4 plays a pivotal role in initiating neuroinflammation. However, the suppressive effects of GA against LPS-induced neuroinflammation and cognitive impairment via TLR4 signaling pathway are not yet well understood. Thus, we hypothesize that GA would prevent LPS-induced inflammatory processes via TLR4 signaling pathway. Our mechanistic studies indicated, for the first time, that the TLR4 signaling pathway was involved in the anti-neuroinflammatory mechanisms of action of GA. Moreover, our results revealed that LPS injection activated the TLR4-mediated inflammatory signaling pathway, indicated by downregulated expressions of Myd88, MAPK, and NF-κB *in* *vivo*. According to the mentioned results, we speculated that GA pretreatment suppressed neuroinflammation by inhibiting the LPS-induced overexpression of IL-1β and TNF-α by targeting TLR4 signaling pathway.

In summary, we have demonstrated that GA, a major active ingredient enriched in the roots of licorice, exerted suppressive effects on LPS-induced neuroinflammation and cognitive impairment in mice. These protective effects were achieved through the inhibition of pro-inflammatory cytokines dependent on TLR4-mediated inflammatory signaling pathway. These results provide further scientific evidence for the health-promoting value of GA products and suggest new applications for medicinal use.
